# Clinical, imaging, and biofluid correlates of Lyme polyradiculitis in a case report of neuroborreliosis

**DOI:** 10.1093/bjrcr/uaaf022

**Published:** 2025-03-20

**Authors:** Michael Tran Duong, Manish Shah, Tatsiana Serhiyenia, Rani Pandya, Ashish Subedi, Charishma Bhimineni, Melissa T Duong, Michelle Heayn, Tanya Ibrahim, Gina Stefanelli, Mudita Patel

**Affiliations:** Department of Medicine, Jefferson Einstein Healthcare Network, Greater Philadelphia, PA 19403, United States; Department of Medicine, University of Pennsylvania Health System, Philadelphia, PA 19104, United States; Department of Medicine, Jefferson Einstein Healthcare Network, Greater Philadelphia, PA 19403, United States; Department of Medicine, Jefferson Einstein Healthcare Network, Greater Philadelphia, PA 19403, United States; Department of Medicine, Jefferson Einstein Healthcare Network, Greater Philadelphia, PA 19403, United States; Department of Medicine, Jefferson Einstein Healthcare Network, Greater Philadelphia, PA 19403, United States; Department of Medicine, Jefferson Einstein Healthcare Network, Greater Philadelphia, PA 19403, United States; Department of Medicine, University of Pennsylvania Health System, Philadelphia, PA 19104, United States; Department of Medicine, Jefferson Einstein Healthcare Network, Greater Philadelphia, PA 19403, United States; Department of Medicine, Jefferson Einstein Healthcare Network, Greater Philadelphia, PA 19403, United States; Department of Medicine, Jefferson Einstein Healthcare Network, Greater Philadelphia, PA 19403, United States; Department of Medicine, Jefferson Einstein Healthcare Network, Greater Philadelphia, PA 19403, United States

**Keywords:** Lyme, MRI, nerve root, neuroimaging, neuroinflammation, polyradiculitis

## Abstract

Among the causes of ambulatory dysfunction, Lyme polyradiculitis is an uncommon but still essential aetiology to consider given its simple, effective treatment. We present a case of a man with 1 month of worsening bilateral leg paresis, paresthesia, and pain. He recalled no erythema migrans or tick bite. Initial screening showed negative serum Lyme and positive Epstein-Barr Virus testing. At our hospital, MRI revealed polyradiculitis with cauda equina nerve root enhancement. Subsequent serum and cerebrospinal results were positive for Lyme neuroborreliosis. He improved rapidly from a course of doxycycline. This case highlights the importance of timing for Lyme serologies in early neuroborreliosis, as well as converging clinical, radiological, and biofluid testing for diagnosis and management.

## Background

Neuroborreliosis is a form of disseminated Lyme disease, an infection caused by *Borrelia burgdorferi*. This spirochete is transmitted to humans by the *Ixodes* tick.^1^ Lyme disease affects hundreds of thousands of Americans every year, but less than 12% experience neurologic deficits.^2^ Prevalence is highest in the summer and fall. Lyme disease progresses from early disease, with symptoms at the first hours to days, and evolves over several months to late disease, which spans over 6 months. Later stages of Lyme disease typically have more disseminated distribution of involvement.

Manifestations of neuroborreliosis involve both the central nervous system (CNS), such as meningitis and encephalomyelitis, and peripheral nervous system, such as polyradiculitis, plexopathy, mononeuropathy, and cranial neuropathy (a form of intracranial peripheral disease).^1^ Peripheral involvement is more prevalent than CNS presentation. In patients with Lyme disease in North America, facial nerve palsy may occur in about 9% (more than 50% of those with neuroborreliosis), followed by radiculitis (4%) and meningitis or encephalitis (1%).^2^ Conversely, in Europe, the most common presentation of CNS Lyme disease is lymphocytic meningoradiculitis (Garin-Bujadoux-Bannwarth syndrome), characterized by sharp, lancinating pain, which may have concurrent cranial neuropathy.^2^^,^^3^ Hence, clinical symptoms should be paired with the clinical context, since isolated Lyme facial nerve palsy is a more common presentation in North America and Lyme polyradiculitis is more common in Europe.

While the manifestations of neuroborreliosis may be protean, the treatment of Lyme disease is straightforward with a course of antibiotics and can lead to rapid resolution of symptoms. Therefore, in patients with suspected neuroborreliosis, is imperative to test serum and cerebrospinal fluid (CSF) Lyme serology, with concurrent imaging and infectious workup. In fact, serum testing alone is not sufficient for the diagnosis of neuroborreliosis. The conjunction of CSF and serum testing allows for appropriate evaluation of *Borrelia* invasion into the nervous system.

Here, we present a case of a man in his 60s who experienced 1 month of progressive leg weakness, pain, and sensory changes. He was admitted to our hospital after worsening bowel and bladder retention. After imaging, serum and CSF testing, a diagnosis of Lyme polyradiculitis was made and appropriate treatment was given. We discuss this case in the context of relevant clinical literature.

## Case presentation

A man in his 60s with hyperlipidaemia, rosacea, prior appendectomy, and cholecystectomy presented in July for 1 month of worsening leg weakness, paresthesias, back pain, constipation, and urinary retention. He was previously active in golf and pickleball, but now had difficulty standing without falling. He initially felt tired after mowing tall grass on his lawn and had small, raised, erythematous rashes on his leg and abdomen, which he attributed to flying insect bites. These self-resolved and were not target-shaped.

**Figure 1.**
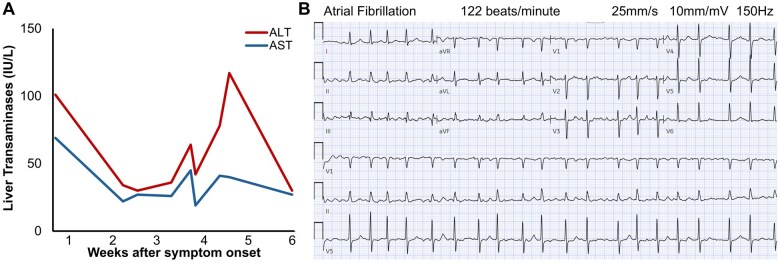
Elevated transaminases and new-onset atrial fibrillation. (A) In this patient with normal baseline liver function presenting 4 weeks after symptom onset, liver enzymes exhibited a temporally biphasic elevation, with ALT > AST. (B) At our hospital, he had new-onset atrial fibrillation with ventricular response rate of 120 to 150 beats/min on electrocardiogram.

He first presented to his primary care provider for sudden-onset fatigue and several days of moderate lumbar back pain. Outpatient screening labs noted high aspartate and alanine transaminases (AST 69, ALT 101, Figure 1A), C-reactive protein (CRP 54), and positive Epstein-Barr Virus (EBV) IgM+/IgG+ antibodies. Viral hepatitis panel was negative. He reported no pets, recent travel, arthralgias or tick bites and Western blot was negative for Lyme serology. Two weeks later, he presented to an outside hospital for abdominal pain. CT imaging of chest, abdomen, and pelvis revealed distension of the bladder and colon without bowel obstruction or enlarged prostate. He had mild leucocytosis but normal AST (22) and ALT (34) (Figure 1A), urinalysis, electrocardiogram, and physical exam so he was discharged with non-steroidal anti-inflammatory medications, laxatives, and scheduled follow-up.

He was admitted to our hospital 1 week later for worsening gait dysfunction, lower back pain, leg weakness, and thigh paresthesias. He reported severe, sharp, electric shock-like pain radiating from the back to his legs, especially at night. Bowel and bladder retention worsened. He had new-onset paroxysmal atrial fibrillation up to 150 beats/min (Figure 1B) that converted to sinus rhythm with intravenous metoprolol tartrate and did not recur. Labs showed elevated AST (41), ALT (78) (Figure 1A), and CRP (10). High-sensitivity troponins were normal and B-Type Natriuretic Peptide (BNP) was 454. Gabapentin 300 mg 3 times daily was started for symptomatic management, but without much relief.

Neurologic examination depicted motor and sensory deficits of the proximal legs with patchy hyporeflexia. Strength of bilateral hip flexors and extensors were 2/5, knee flexors/extensors were 4/5, and foot dorsiflexors and plantarflexors were 5/5. Medial and lateral thighs demonstrated decreased sensation bilaterally to light touch, pinprick, temperature, vibration, and proprioception. Sensation in the lower legs and feet was intact. Patellar reflexes were 1+ bilaterally, while ankle reflex was 1+ on the left and 0 on the right. Heal-to-shin manoeuvre was poor. Gait was unsteady and broad. Romberg sign was positive, suggesting an abnormality of proprioception in the spinal cord and/or vestibular sense. The neck and arms were normal in strength and sensation. Upper limbs had no dysmetria, dysdiadochokinesia, or cogwheel rigidity. No Babinski sign, Hoffmann sign, or abnormal ankle clonus was seen. Nuchal rigidity and Kernig signs were not found. Cranial nerve and cognitive assessment was normal.

**Figure 2.**
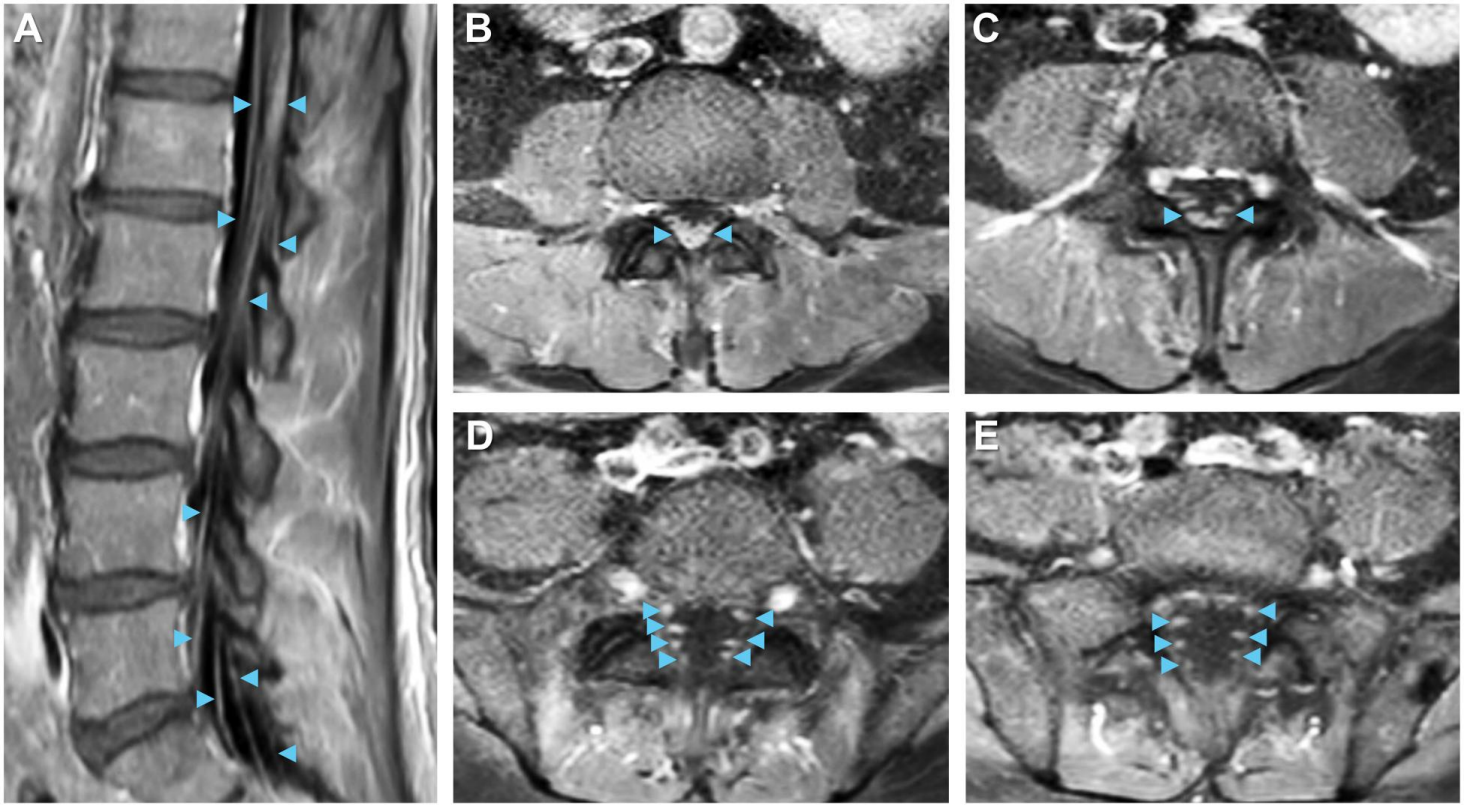
Neuroimaging visualizes lumbar polyradiculitis. Enhancement of bilateral cauda equina nerve roots (arrowheads) near L1-L5 on (A) sagittal and (B-E) descending axial slices supports the diagnosis of lumbar polyradiculitis on T1 post-contrast MRI.

**Figure 3.**
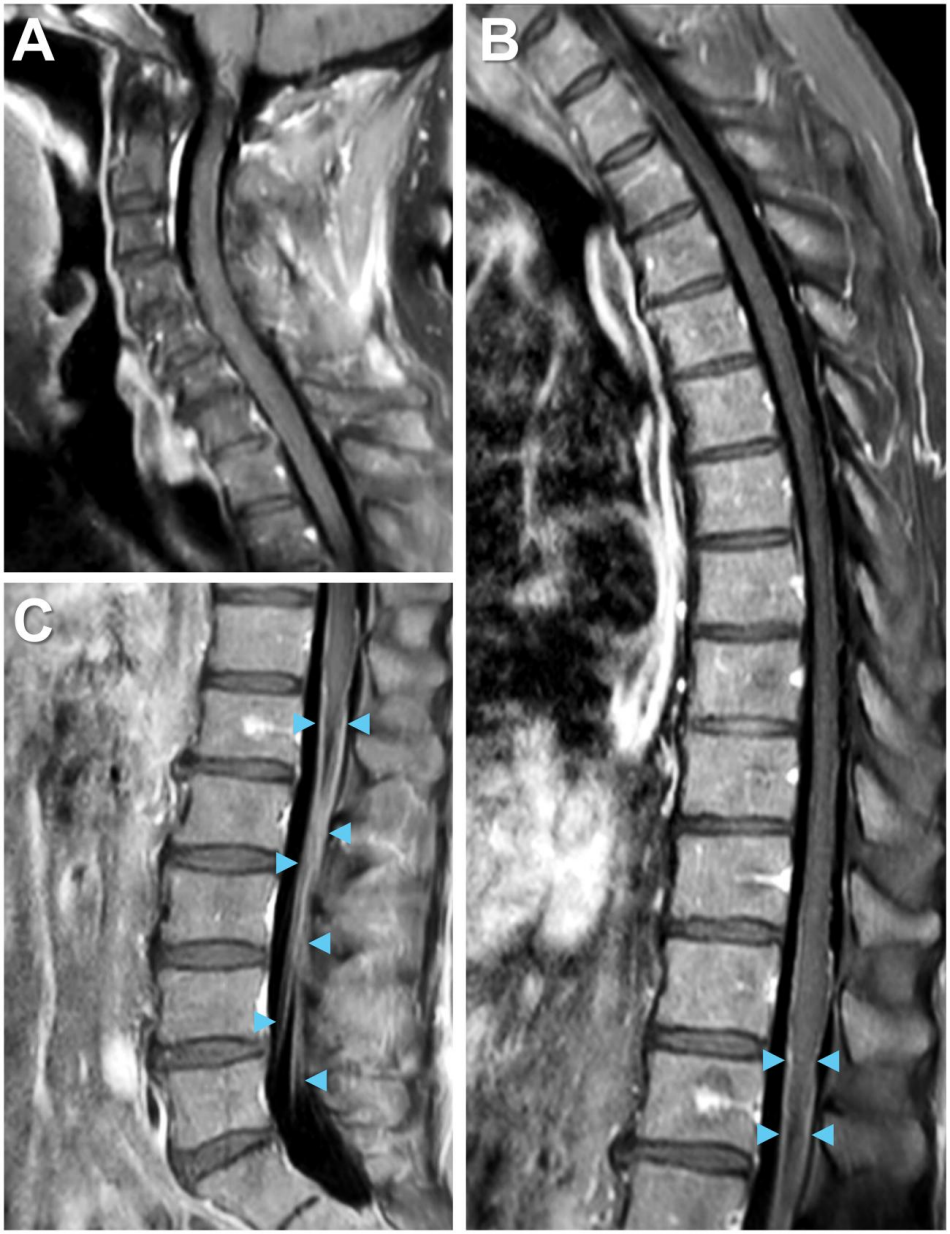
Complete spine imaging on MRI. The majority of contrast enhancement is not readily seen in the (A) cervical or (B) thoracic but rather the (C) lumbar region on T1 post-contrast spine imaging. Enhancement of bilateral cauda equina nerve roots (arrowheads).

**Figure 4.**
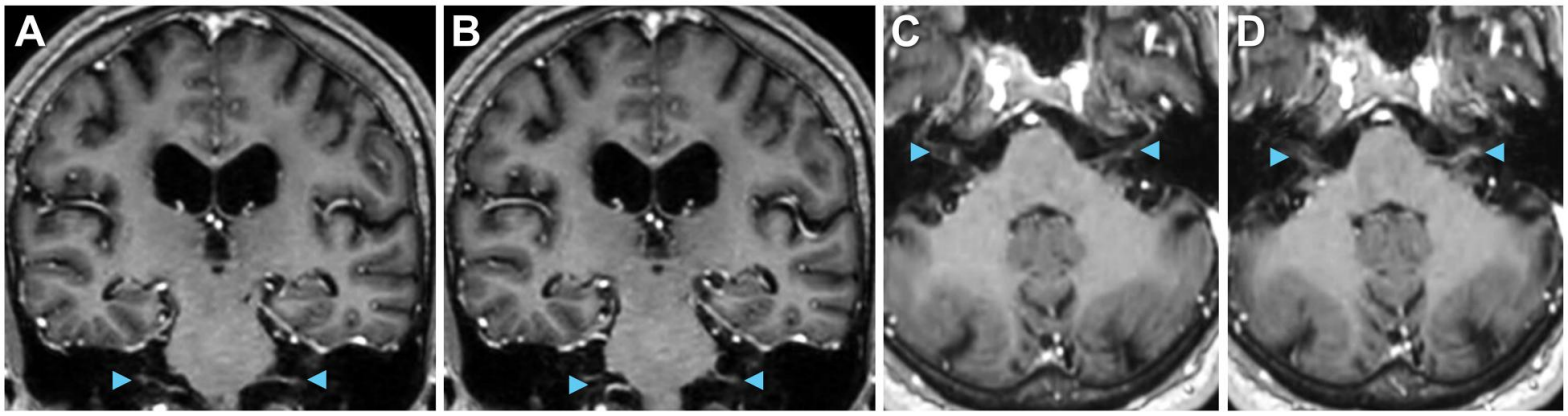
Cranial nerves on MRI. Suggestion of cranial nerve involvement (arrowheads), including cranial nerve V on (A and B) coronal and (C and D) axial T1 post-contrast MRI.

Total spine MRI with gadolinium-based contrast revealed enhancement of cauda equina nerve roots at the levels of L1 to L5 (Figure 2A-D) without abnormalities of the spinal cord, neural foramina, or vertebrae, indicating lumbosacral polyradiculitis. Complete spine MRI demonstrated that the majority of contrast enhancement was limited to the lumbar region (Figure 3). Brain MRI with contrast suggested cranial nerve VII enhancement bilaterally (Figure 4). Additional serum tests were negative. Vitamin B12, B9, B6, B1, zinc, copper, anti-nuclear antibody levels, and erythrocyte sedimentation rate were normal.

**Figure 5.**
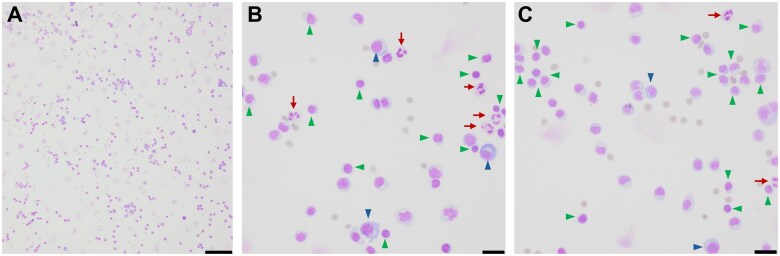
CSF cytology illustrates lymphocytic pleocytosis. Air-dried hematoxylin-eosin stained CSF micrographs at (A) 10× (scale bar 100 μm) and (B and C) 40× (scale bar 20 μm) indicate lymphocytic pleocytosis (arrows) with abundant polymorphous lymphocytes (green arrowheads) and plasma cells (blue arrowheads) with occasional neutrophils (red arrows).

Lumbar puncture was performed. Initial CSF testing showed normal glucose (67), elevated protein (149), and pleocytosis (114) of lymphocyte predominance (Figure 5). CSF non-specific IgG was high and multiple oligoclonal bands were present. Differential diagnosis included polyradiculitis due to neuroborreliosis, neurosyphilis, flaviviruses, enteroviruses, and other tick-borne illnesses as well as atypical post-infectious acute inflammatory demyelinating polyradiculopathy (AIDP) on the Guillain-Barré spectrum or less likely to be chronic inflammatory demyelinating polyradiculoneuropathy (CIDP) or neurolymphomatosis. A full differential diagnosis of nerve root enhancement is further summarized in Table 1. Empiric doxycycline 100 mg twice daily was started and the patient experienced reduction in lumbar pain within 2 doses.

**Table 1.** Differential diagnosis for nerve root enhancement on MRI.

Aetiology	Disease
Immunological	AIDP, CIDP, neurosarcoidosis
Infection	Lyme disease, syphilis, HIV, HSV, VZV, CMV, poliomyelitis, tickborne encephalitis virus, leptomeningitis
Neoplasm	Leptomeningeal metastasis, lymphomatous infiltration
Trauma	Traumatic injury, post-operative
Genetic	Charcot-Marie-Tooth syndrome, lysosomal storage disorders

Microbiological labs resulted. Notably, serum Western blot was now Lyme IgM+ (2/3 reactive bands) and IgG– (only 4/10 reactive bands). Serum testing was negative for Human Immunodeficiency Virus (HIV), West Nile Virus (WNV), syphilis, *Anaplasma*, and *Ehrlichia*. Peripheral smear showed no *Babesia*, *Malaria*, or other intracellular parasites. CSF immunoassay exhibited Lyme IgM+ and IgG+ antibodies. CSF was negative for WNV, Herpes Simplex Virus (HSV) 1 and 2, Varicella Zoster Virus (VZV), Cytomegalovirus (CMV), Human Herpesvirus 6 (HHV6), enterovirus, *Streptococcus pneumoniae*, *Neisseria meningitidis*, *Haemophilus influenzae*, *Listeria monocytogenes*, *Escherichia coli*, syphilis, and *Cryptococcus*.

Therapeutic doxycycline 100 mg twice daily was continued for a 21-day course. Lumbar pain, leg paresthesias, and urinary retention were alleviated within 2 days of antibiotics and he quickly regained strength to ambulate steadily. Transaminase levels normalized within 2 weeks (Figure 1A). At a physical therapy centre 1 month after discharge, he demonstrated improved ability to perform chores and walk around the house. At 4-month follow-up, the patient had resolved dysesthesias and substantial recovery to baseline in recreational sports, exercise and activities of daily living.

## Discussion

This case highlights a patient with early neuroborreliosis with leg paresis, pain, paresthesias, and bowel/bladder retention. Lyme polyradiculitis is less common in the United States than in Europe but should still be considered on a differential diagnosis. This demonstrates a role of imaging and serology testing/retesting for patients with suspected secondary Lyme disease, even without classic erythema migrans, recognized tick bite or recent travel. While initial Lyme screening at 1 week after likely exposure was negative, repeat testing at 4 weeks was IgM+ in serum and IgM+/IgG+ in CSF. EBV serology can be falsely positive in early disseminated Lyme disease due to epitope cross reactivity,^4^ which may have been the case here.

Our case emphasizes the correlation between clinical signs, imaging, and serum/CSF tests. Cranial neuropathy is considered the most common neurological presentation of Lyme disease in North America,^2^ while polyradiculitis is the most frequent phenotype in CNS Lyme disease in Europe,^3^ though this prevalence may be different in North America. Here, the primary symptom is lower back pain, as well as bowel and bladder incontinence. Given this patient’s presentation, Lyme polyradiculitis can roughly mimic AIDP, as both lead to nerve root enhancement on MRI and both exhibit lower motor neuron signs including painful dysesthesias, hyporeflexia, and bowel/bladder retention.^5^ However, AIDP shows CSF albuminocytologic dissociation,^6^ while Lyme polyradiculitis has dually elevated CSF protein and white blood cells (WBCs). CSF pleocytosis in Lyme disease may be enriched for lymphocytes and/or monocytes.^1^ Lyme neuroborreliosis can commonly shows cranial nerve enhancement on MRI or cranial neuropathy on physical exam. In contrast, classic sensorimotor AIDP carries the risk of progression to respiratory failure and typically does not involve the cranial nerves except in bulbar and facial variants of AIDP, including Miller-Fisher syndrome and Bickerstaff brainstem encephalitis. These rare, atypical bulbar variants on the Guillain-Barré spectrum are linked to anti-ganglioside GQ1b or GT1a serum autoantibodies.^2^^,^^5^ Generally, neuroborreliosis has a varied imaging presentation, from normal brain MRI to nerve and meningeal enhancement to vasculitis, infarct and haemorrhage,^7^ though Lyme polyradiculitis is best visualized by nerve root enhancement^5^ (Table 1).

Patients with early disseminated Lyme disease can sustain mild liver injury secondary to spirochete infiltration of hepatic parenchyma and sinusoids, though co-infection with other tick-borne infections can also incite transaminase elevation.^8^ ALT can be higher than AST in Lyme disease,^9^ as seen in our patient. Specifically, the patient had a temporally biphasic transaminase spike, which can occur during the progression of disseminated tick-borne and zoonotic infections. Note that elevated transaminases can reflect both Lyme-associated hepatitis and myositis.^10^

Disseminated Lyme can cause carditis leading to arrhythmia.^1^ Our patient had new-onset paroxysmal atrial fibrillation but did not develop Lyme carditis. Subsequent PR intervals were normal (160 ms) with unremarkable serial troponins and transthoracic echocardiogram (except for left ventricular concentric hypertrophy, likely chronic). Nuclear stress test at 2 weeks after hospitalization was negative for abnormal myocardial perfusion. Thus, the new-onset atrial fibrillation may have been secondary to systemic illness.

Typical treatment for neuroborreliosis is 14-21 days of oral doxycycline, intravenous ceftriaxone, or penicillin.^1^^,^^2^^,^^10^ Oral doxycycline was demonstrated to be equally effective as penicillin and ceftriaxone,^1^^,^^10^ so doxycycline is often preferred due to improved compliance. Studies show nearly 100% response rate to doxycycline, though a proportion of patients (20%-60%) may experience some residual neurologic deficits 1 year later.^1^^,^^10^ Current clinical guidelines do not provide specific recommendations about corticosteroids for CNS Lyme manifestations due to limited evidence of efficacy.^1^ This ongoing debate, particularly for Lyme facial nerve palsy and Lyme arthritis, is a future direction of inquiry.

Overall, polyradiculitis from Lyme neuroborreliosis can be considered in patients with leg weakness, painful paresthesia, elevated transaminases, and nerve root enhancement on MRI, including in patients without classic rash or initial positive Lyme serology. Clinicians are encouraged to keep a high index of suspicion in patients presenting with ambulatory dysfunction and additional systemic symptoms to ensure thoughtful differential diagnoses, such as Lyme polyradiculitis, are considered within the appropriate diagnostic and clinical context.

## Learning points

- Lyme polyradiculitis is an uncommon but important cause of ambulatory dysfunction and back pain, especially given its straigtforward management and treatment response.
- Spine MRI with contrast reveals nerve root enhancement in Lyme polyradiculitis. Brain MRI with contrast can also highlight cranial nerve involvement.
- Serum studies can be negative for Lyme serology early on in Lyme disease course. Cerebrospinal fluid studies are imperative for the diagnosis of neuroborreliosis.
